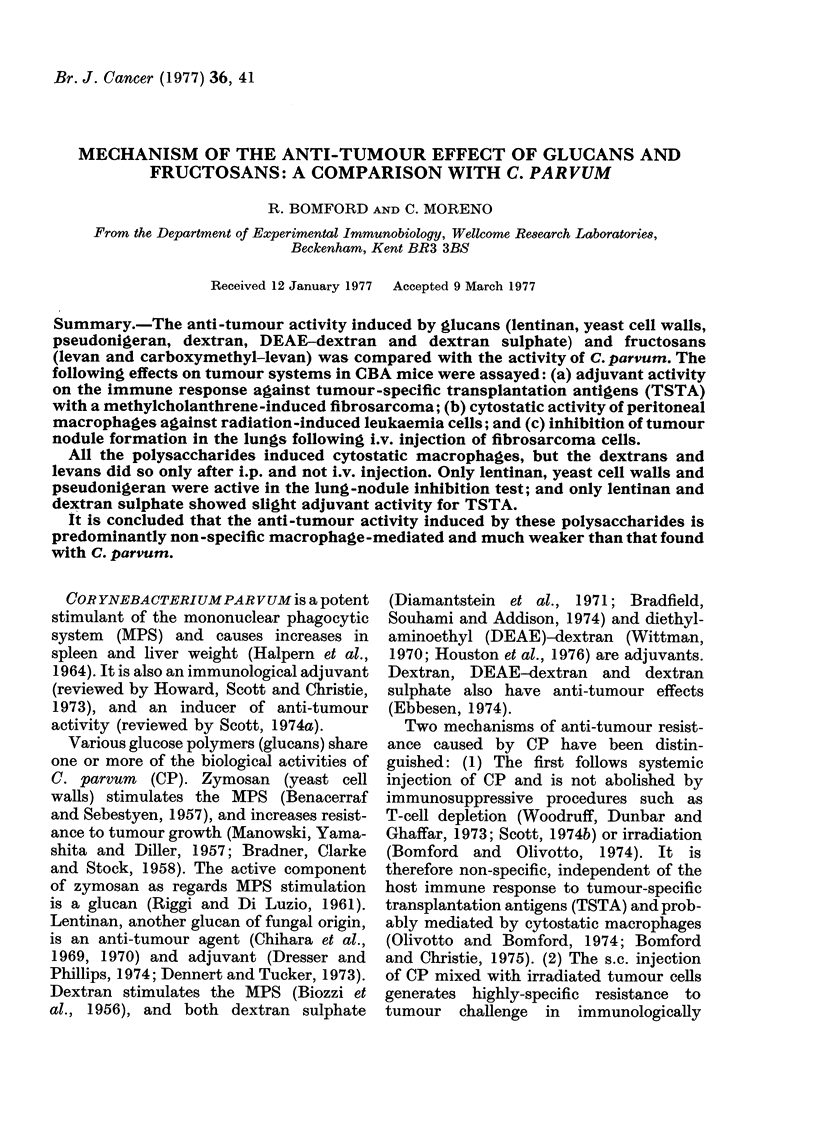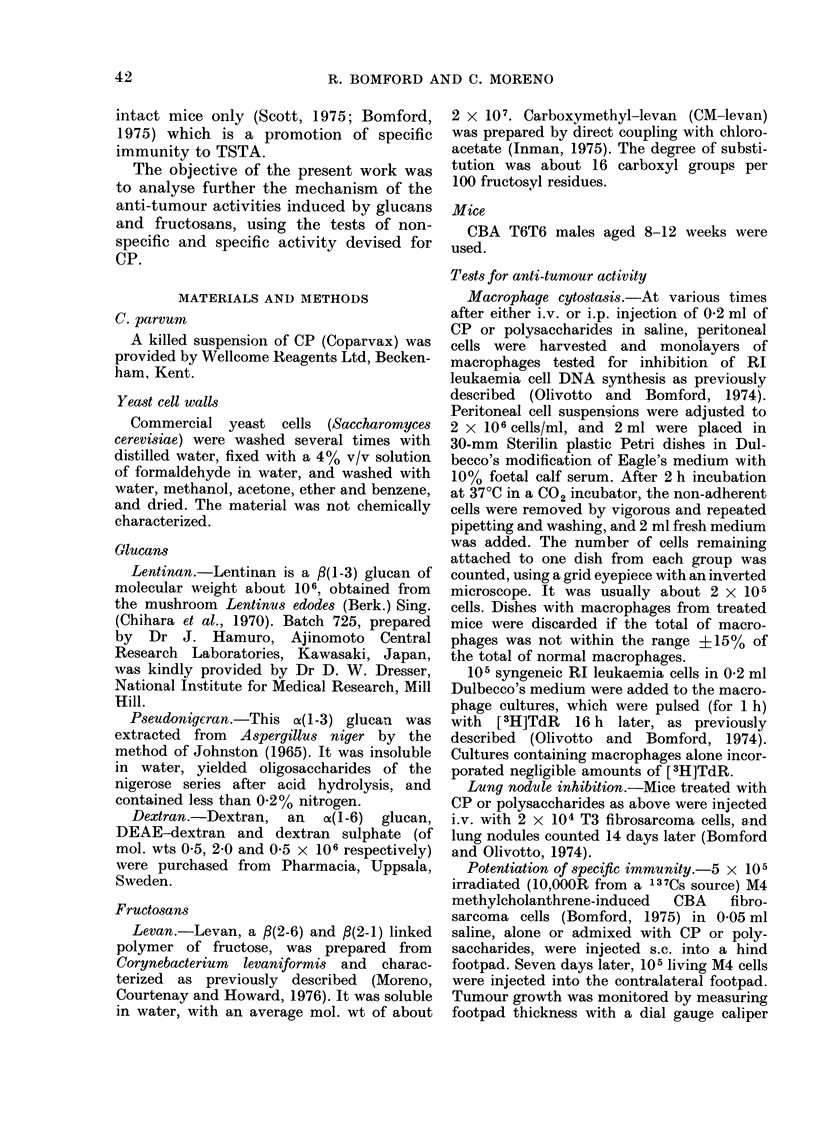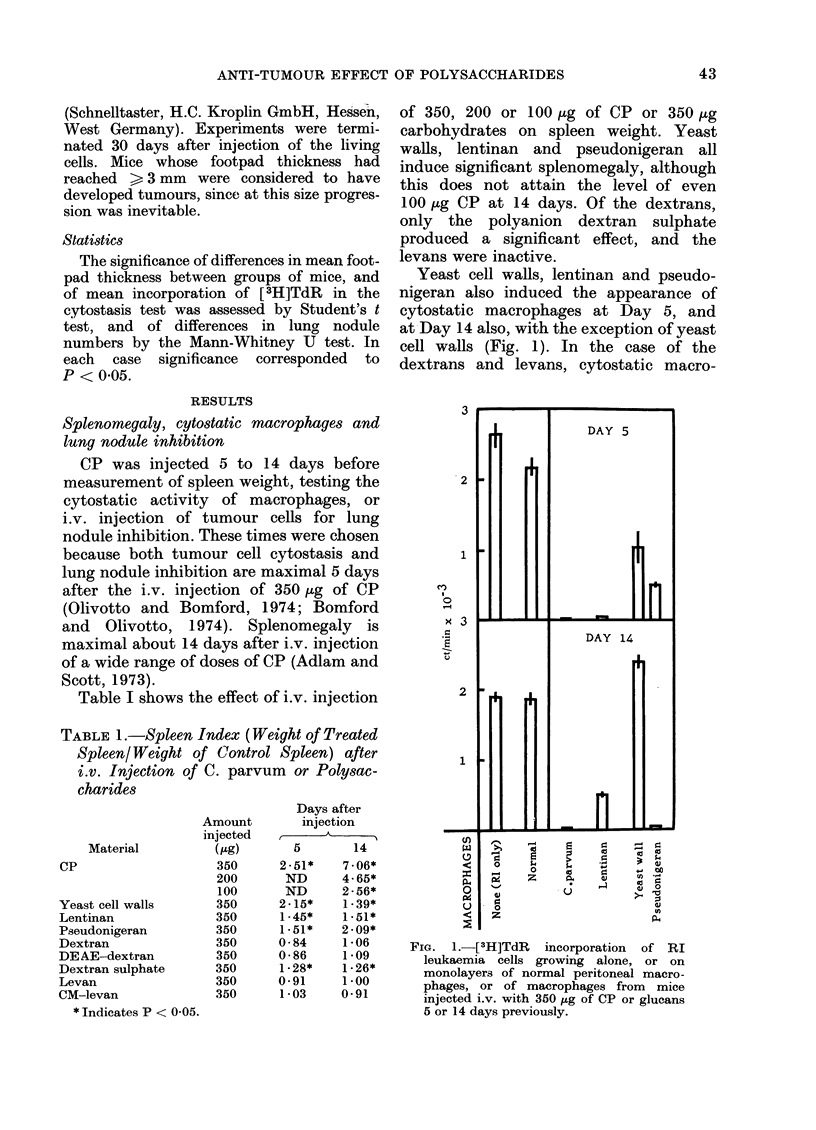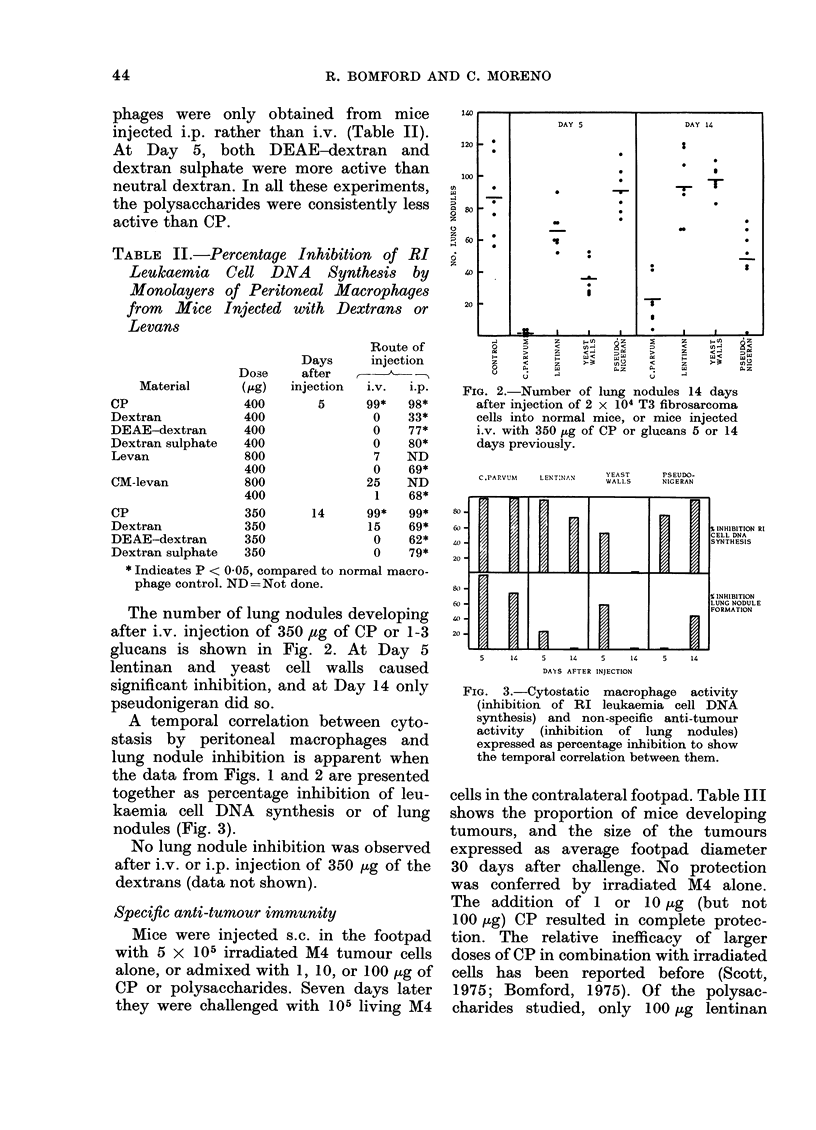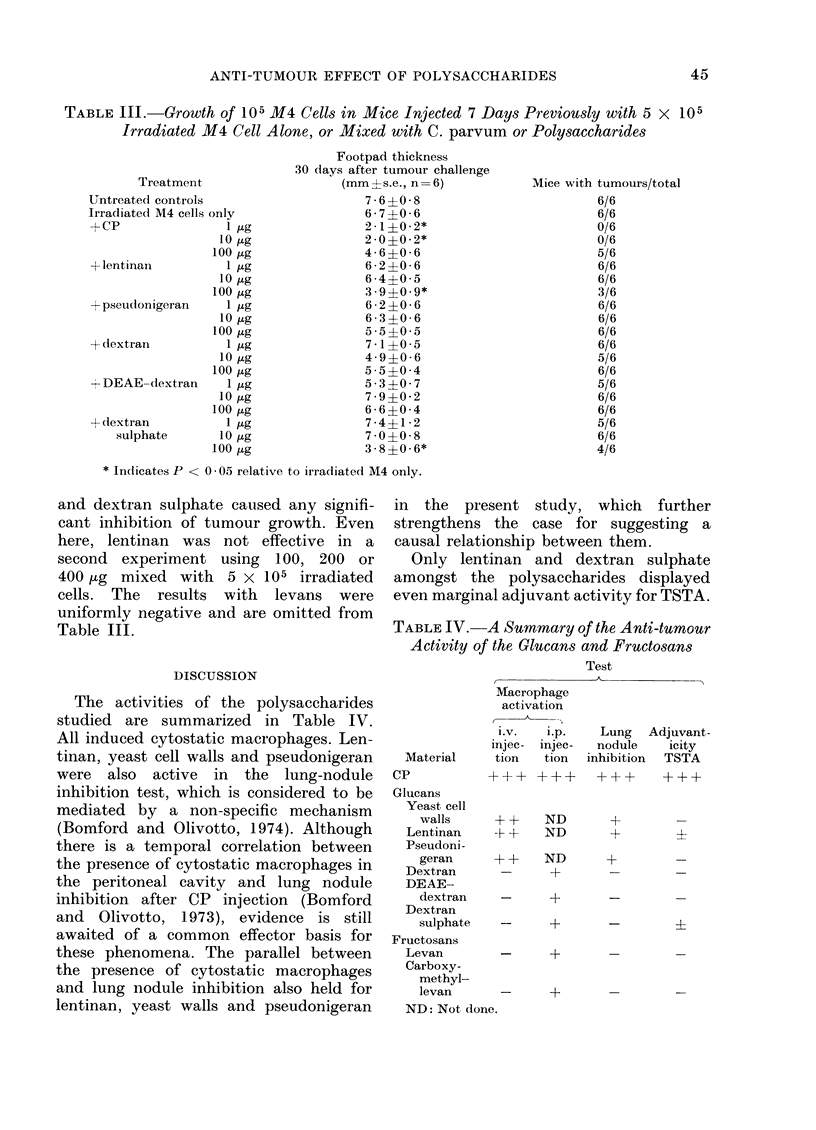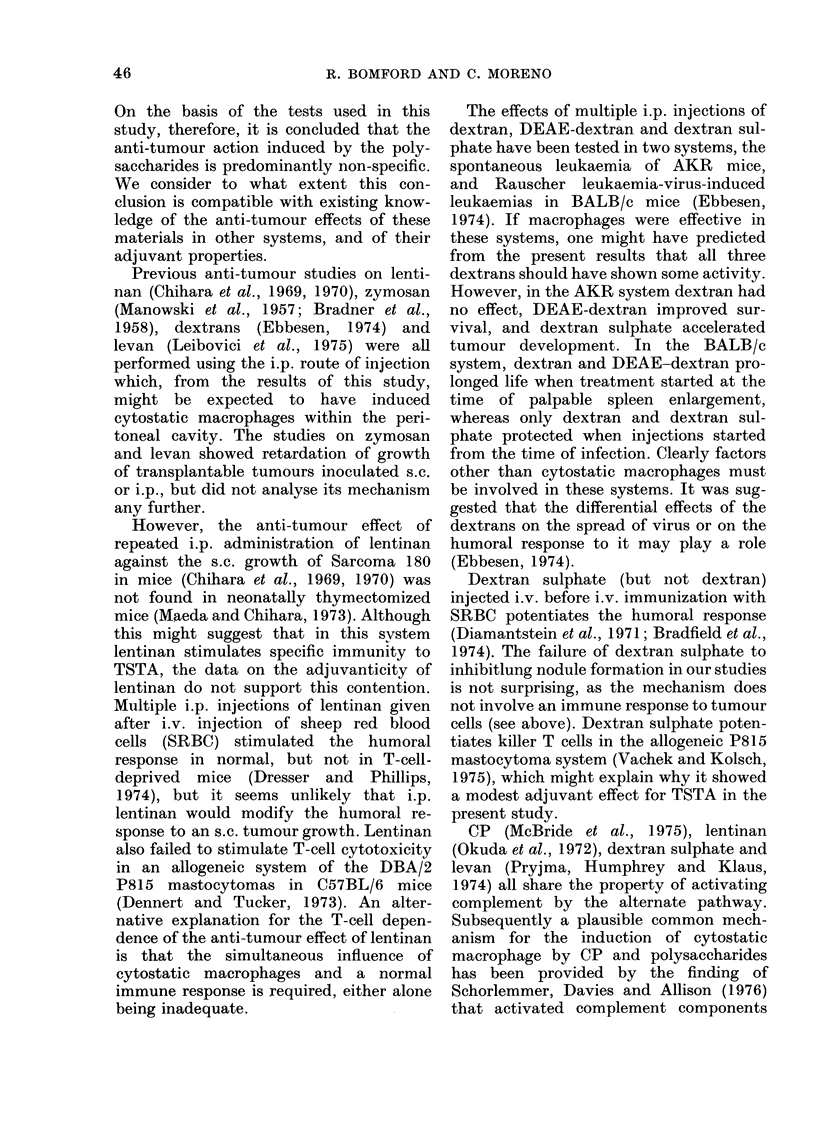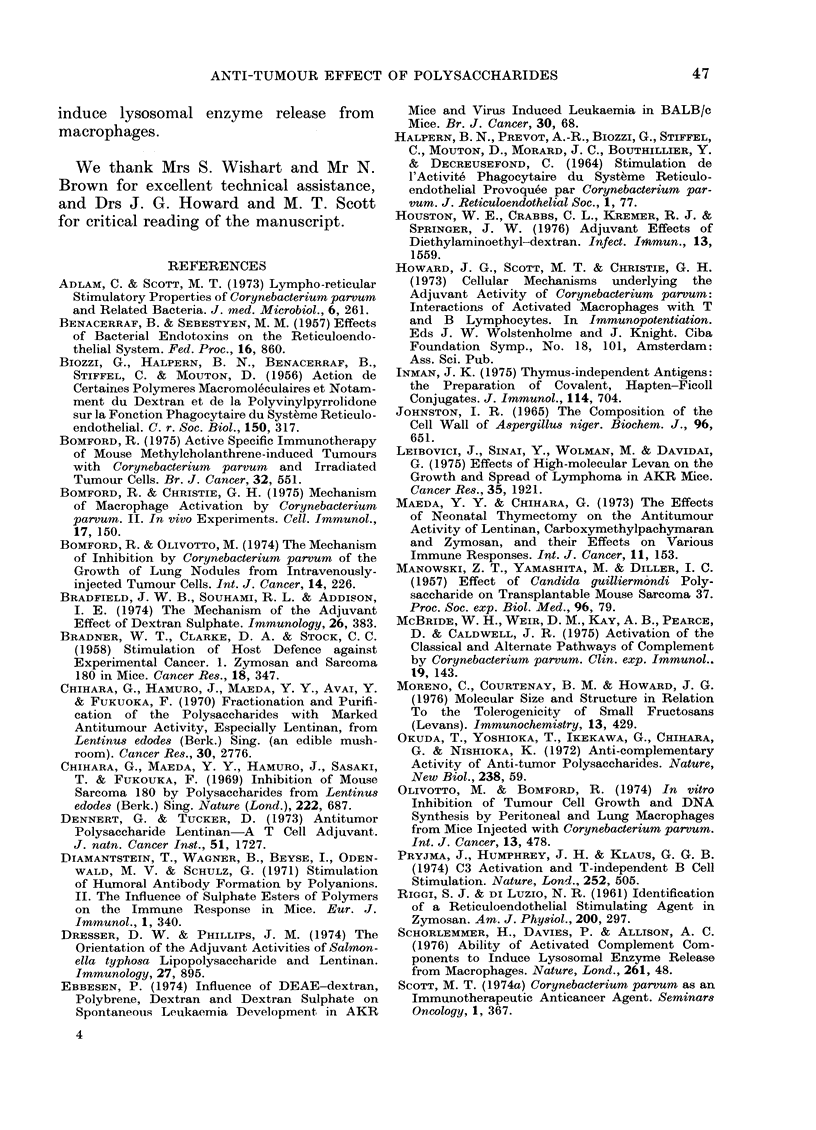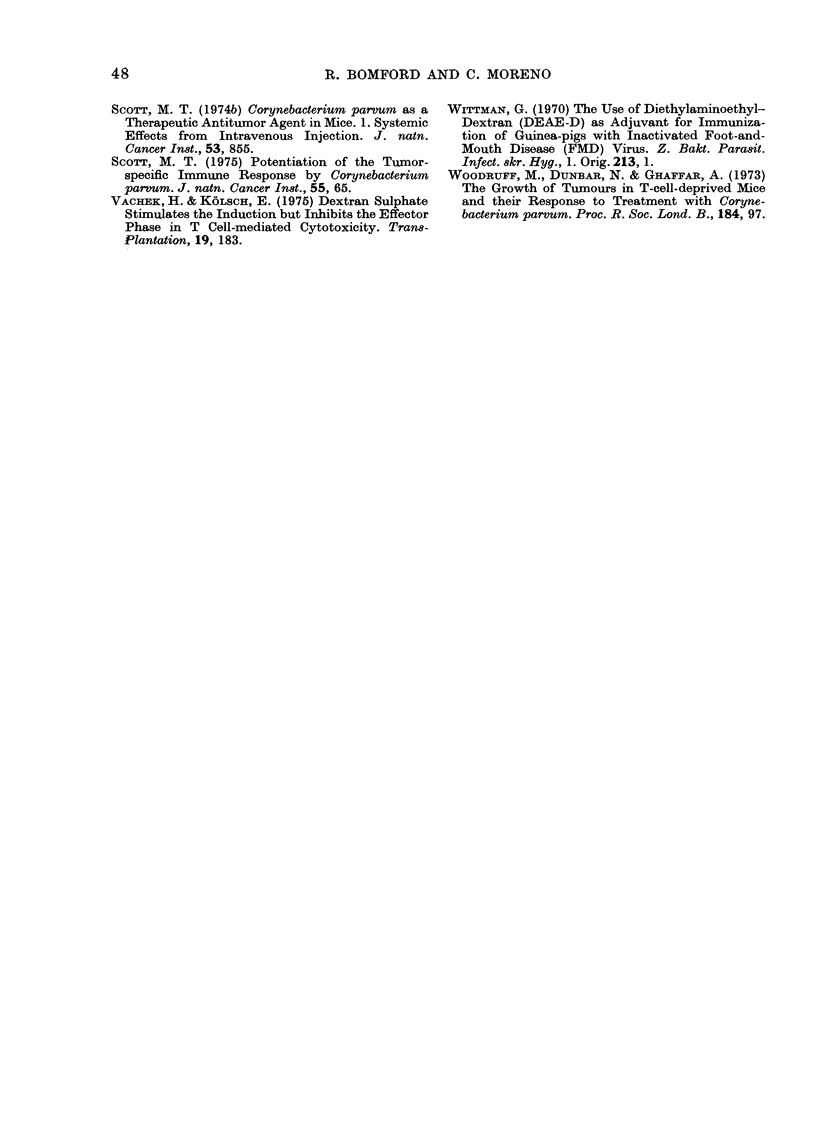# Mechanism of the anti-tumour effect of glucans and fructosans: a comparison with C. parvum.

**DOI:** 10.1038/bjc.1977.152

**Published:** 1977-07

**Authors:** R. Bomford, C. Moreno

## Abstract

The anti-tumour activity induced by glucans (lentinan, yeast cell walls, pseudonigeran, dextran, DEAE-dextran and dextran sulphate) and fructosans (levan and carboxymethyl-levan) was compared with the activity of C. parvum. The following effects on tumour systems in CBA mice were assayed: (a) adjuvant activity on the immune response against tumour-specific transplantation antigens (TSTA) with a methylcholanthrene-induced fibrosarcoma; (b) cytostatic activity of peritoneal macrophages against radiation-induced leukaemia cells; and (c) inhibition of tumour nodule formation in the lungs following i.v. injection of fibrosarcoma cells. All the polysaccharides induced cytostatic macrophages, but the dextrans and levans did so only after i.p. and not i.v. injection. Only lentinan, yeast cell walls and pseudonigeran were active in the lung-nodule inhibition test; and only lentinan and dextran sulphate showed slight adjuvant activity for TSTA. It is concluded that the anti-tumour activity induced by these polysaccharides is predominantly non-specific macrophage-mediated and much weaker than that found with C. parvum.


*Br. J. Cancer* (1977) **36**, 41

### MECHANISM OF THE ANTI-TUMOUR EFFECT OF GLUCANS AND FRUCTOSANS: A COMPARISON WITH *C. PARVUM*

R. BOMFORD AND C. MORENO

*From the Department of Experimental Immunobiology, Wellcome Research Laboratories, Beckenham, Kent BR3 3BS*

Received 12 January 1977 Accepted 9 March 1977

**Summary.—The anti-tumour activity induced by glucans (lentinan, yeast cell walls, pseudonigeran, dextran, DEAE–dextran and dextran sulphate) and fructosans (levan and carboxymethyl–levan) was compared with the activity of *C. parvum*. The following effects on tumour systems in CBA mice were assayed: (a) adjuvant activity on the immune response against tumour-specific transplantation antigens (TSTA) with a methylcholanthrene-induced fibrosarcoma; (b) cytostatic activity of peritoneal macrophages against radiation-induced leukaemia cells; and (c) inhibition of tumour nodule formation in the lungs following i.v. injection of fibrosarcoma cells.**

**All the polysaccharides induced cytostatic macrophages, but the dextrans and levans did so only after i.p. and not i.v. injection. Only lentinan, yeast cell walls and pseudonigeran were active in the lung-nodule inhibition test; and only lentinan and dextran sulphate showed slight adjuvant activity for TSTA.**

**It is concluded that the anti-tumour activity induced by these polysaccharides is predominantly non-specific macrophage-mediated and much weaker than that found with *C. parvum*.**

*CORYNEBACTERIUM PARVUM* is a potent stimulant of the mononuclear phagocytic system (MPS) and causes increases in spleen and liver weight (Halpern *et al.*, 1964). It is also an immunological adjuvant (reviewed by Howard, Scott and Christie, 1973), and an inducer of anti-tumour activity (reviewed by Scott, 1974*a*).

Various glucose polymers (glucans) share one or more of the biological activities of *C. parvum* (CP). Zymosan (yeast cell walls) stimulates the MPS (Benacerraf and Sebestyen, 1957), and increases resistance to tumour growth (Manowski, Yamashita and Diller, 1957; Bradner, Clarke and Stock, 1958). The active component of zymosan as regards MPS stimulation is a glucan (Riggi and Di Luzio, 1961). Lentinan, another glucan of fungal origin, is an anti-tumour agent (Chihara *et al.*, 1969, 1970) and adjuvant (Dresser and Phillips, 1974; Dennert and Tucker, 1973). Dextran stimulates the MPS (Biozzi *et al.*, 1956), and both dextran sulphate (Diamantstein *et al.*, 1971; Bradfield, Souhami and Addison, 1974) and diethylaminoethyl (DEAE)–dextran (Wittman, 1970; Houston *et al.*, 1976) are adjuvants. Dextran, DEAE–dextran and dextran sulphate also have anti-tumour effects (Ebbesen, 1974).

Two mechanisms of anti-tumour resistance caused by CP have been distinguished: (1) The first follows systemic injection of CP and is not abolished by immunosuppressive procedures such as T-cell depletion (Woodruff, Dunbar and Ghaffar, 1973; Scott, 1974*b*) or irradiation (Bomford and Olivotto, 1974). It is therefore non-specific, independent of the host immune response to tumour-specific transplantation antigens (TSTA) and probably mediated by cytostatic macrophages (Olivotto and Bomford, 1974; Bomford and Christie, 1975). (2) The s.c. injection of CP mixed with irradiated tumour cells generates highly-specific resistance to tumour challenge in immunologically intact mice only (Scott, 1975; Bomford, 1975) which is a promotion of specific immunity to TSTA.

The objective of the present work was to analyse further the mechanism of the anti-tumour activities induced by glucans and fructosans, using the tests of non-specific and specific activity devised for CP.

### MATERIALS AND METHODS

### *C. parvum*

A killed suspension of CP (Coparvax) was provided by Wellcome Reagents Ltd, Beckenham, Kent.

### *Yeast cell walls*

Commercial yeast cells (*Saccharomyces cerevisiae*) were washed several times with distilled water, fixed with a 4% v/v solution of formaldehyde in water, and washed with water, methanol, acetone, ether and benzene, and dried. The material was not chemically characterized.

### *Glucans*

*Lentinan.*—Lentinan is a $\beta$(1-3) glucan of molecular weight about $10^6$, obtained from the mushroom *Lentinus edodes* (Berk.) Sing. (Chihara *et al.*, 1970). Batch 725, prepared by Dr J. Hamuro, Ajinomoto Central Research Laboratories, Kawasaki, Japan, was kindly provided by Dr D. W. Dresser, National Institute for Medical Research, Mill Hill.

*Pseudonigeran.*—This $\alpha$(1-3) glucan was extracted from *Aspergillus niger* by the method of Johnston (1965). It was insoluble in water, yielded oligosaccharides of the nigerose series after acid hydrolysis, and contained less than 0·2% nitrogen.

*Dextran.*—Dextran, an $\alpha$(1-6) glucan, DEAE–dextran and dextran sulphate (of mol. wts 0·5, 2·0 and 0·5 $\times$ $10^6$ respectively) were purchased from Pharmacia, Uppsala, Sweden.

### *Fructosans*

*Levan.*—Levan, a $\beta$(2-6) and $\beta$(2-1) linked polymer of fructose, was prepared from *Corynebacterium levaniformis* and characterized as previously described (Moreno, Courtenay and Howard, 1976). It was soluble in water, with an average mol. wt of about 2 $\times$ $10^7$. Carboxymethyl–levan (CM–levan) was prepared by direct coupling with chloroacetate (Inman, 1975). The degree of substitution was about 16 carboxyl groups per 100 fructosyl residues.

### *Mice*

CBA T6T6 males aged 8–12 weeks were used.

### *Tests for anti-tumour activity*

*Macrophage cytostasis.*—At various times after either i.v. or i.p. injection of 0·2 ml of CP or polysaccharides in saline, peritoneal cells were harvested and monolayers of macrophages tested for inhibition of RI leukaemia cell DNA synthesis as previously described (Olivotto and Bomford, 1974). Peritoneal cell suspensions were adjusted to 2 $\times$ $10^6$ cells/ml, and 2 ml were placed in 30-mm Sterilin plastic Petri dishes in Dulbecco's modification of Eagle's medium with 10% foetal calf serum. After 2 h incubation at 37°C in a $CO_2$ incubator, the non-adherent cells were removed by vigorous and repeated pipetting and washing, and 2 ml fresh medium was added. The number of cells remaining attached to one dish from each group was counted, using a grid eyepiece with an inverted microscope. It was usually about 2 $\times$ $10^5$ cells. Dishes with macrophages from treated mice were discarded if the total of macrophages was not within the range $\pm$15% of the total of normal macrophages.

$10^5$ syngeneic RI leukaemia cells in 0·2 ml Dulbecco's medium were added to the macrophage cultures, which were pulsed (for 1 h) with [$^3$H]TdR 16 h later, as previously described (Olivotto and Bomford, 1974). Cultures containing macrophages alone incorporated negligible amounts of [$^3$H]TdR.

*Lung nodule inhibition.*—Mice treated with CP or polysaccharides as above were injected i.v. with 2 $\times$ $10^4$ T3 fibrosarcoma cells, and lung nodules counted 14 days later (Bomford and Olivotto, 1974).

*Potentiation of specific immunity.*—5 $\times$ $10^5$ irradiated (10,000R from a $^{137}$Cs source) M4 methylcholanthrene-induced CBA fibrosarcoma cells (Bomford, 1975) in 0·05 ml saline, alone or admixed with CP or polysaccharides, were injected s.c. into a hind footpad. Seven days later, $10^5$ living M4 cells were injected into the contralateral footpad. Tumour growth was monitored by measuring footpad thickness with a dial gauge caliper

(Schnelltaster, H.C. Kroplin GmbH, Hessen, West Germany). Experiments were terminated 30 days after injection of the living cells. Mice whose footpad thickness had reached $\geqslant 3$ mm were considered to have developed tumours, since at this size progression was inevitable.

*Statistics*

The significance of differences in mean footpad thickness between groups of mice, and of mean incorporation of [$^3$H]TdR in the cytostasis test was assessed by Student's *t* test, and of differences in lung nodule numbers by the Mann-Whitney U test. In each case significance corresponded to $P < 0{\cdot}05$.

### RESULTS

*Splenomegaly, cytostatic macrophages and lung nodule inhibition*

CP was injected 5 to 14 days before measurement of spleen weight, testing the cytostatic activity of macrophages, or i.v. injection of tumour cells for lung nodule inhibition. These times were chosen because both tumour cell cytostasis and lung nodule inhibition are maximal 5 days after the i.v. injection of 350 μg of CP (Olivotto and Bomford, 1974; Bomford and Olivotto, 1974). Splenomegaly is maximal about 14 days after i.v. injection of a wide range of doses of CP (Adlam and Scott, 1973).

Table I shows the effect of i.v. injection of 350, 200 or 100 μg of CP or 350 μg carbohydrates on spleen weight. Yeast walls, lentinan and pseudonigeran all induce significant splenomegaly, although this does not attain the level of even 100 μg CP at 14 days. Of the dextrans, only the polyanion dextran sulphate produced a significant effect, and the levans were inactive.

Yeast cell walls, lentinan and pseudonigeran also induced the appearance of cytostatic macrophages at Day 5, and at Day 14 also, with the exception of yeast cell walls (Fig. 1). In the case of the dextrans and levans, cytostatic macro-

TABLE 1.—*Spleen Index (Weight of Treated Spleen/Weight of Control Spleen) after i.v. Injection of* C. parvum *or Polysaccharides*

| Material | Amount injected (μg) | Days after injection 5 | 14 |
|---|---|---|---|
| CP | 350 | 2·51* | 7·06* |
| | 200 | ND | 4·65* |
| | 100 | ND | 2·56* |
| Yeast cell walls | 350 | 2·15* | 1·39* |
| Lentinan | 350 | 1·45* | 1·51* |
| Pseudonigeran | 350 | 1·51* | 2·09* |
| Dextran | 350 | 0·84 | 1·06 |
| DEAE–dextran | 350 | 0·86 | 1·09 |
| Dextran sulphate | 350 | 1·28* | 1·26* |
| Levan | 350 | 0·91 | 1·00 |
| CM–levan | 350 | 1·03 | 0·91 |

* Indicates $P < 0{\cdot}05$.

FIG. 1.—[$^3$H]TdR incorporation of RI leukaemia cells growing alone, or on monolayers of normal peritoneal macrophages, or of macrophages from mice injected i.v. with 350 μg of CP or glucans 5 or 14 days previously.

phages were only obtained from mice injected i.p. rather than i.v. (Table II). At Day 5, both DEAE–dextran and dextran sulphate were more active than neutral dextran. In all these experiments, the polysaccharides were consistently less active than CP.

TABLE II.—*Percentage Inhibition of RI Leukaemia Cell DNA Synthesis by Monolayers of Peritoneal Macrophages from Mice Injected with Dextrans or Levans*

| Material | Dose ($\mu$g) | Days after injection | Route of injection i.v. | Route of injection i.p. |
|---|---|---|---|---|
| CP | 400 | 5 | 99* | 98* |
| Dextran | 400 | | 0 | 33* |
| DEAE–dextran | 400 | | 0 | 77* |
| Dextran sulphate | 400 | | 0 | 80* |
| Levan | 800 | | 7 | ND |
| | 400 | | 0 | 69* |
| CM-levan | 800 | | 25 | ND |
| | 400 | | 1 | 68* |
| CP | 350 | 14 | 99* | 99* |
| Dextran | 350 | | 15 | 69* |
| DEAE–dextran | 350 | | 0 | 62* |
| Dextran sulphate | 350 | | 0 | 79* |

* Indicates $P < 0{\cdot}05$, compared to normal macrophage control. ND=Not done.

The number of lung nodules developing after i.v. injection of 350 $\mu$g of CP or 1-3 glucans is shown in Fig. 2. At Day 5 lentinan and yeast cell walls caused significant inhibition, and at Day 14 only pseudonigeran did so.

A temporal correlation between cytostasis by peritoneal macrophages and lung nodule inhibition is apparent when the data from Figs. 1 and 2 are presented together as percentage inhibition of leukaemia cell DNA synthesis or of lung nodules (Fig. 3).

No lung nodule inhibition was observed after i.v. or i.p. injection of 350 $\mu$g of the dextrans (data not shown).

*Specific anti-tumour immunity*

Mice were injected s.c. in the footpad with 5 × $10^5$ irradiated M4 tumour cells alone, or admixed with 1, 10, or 100 $\mu$g of CP or polysaccharides. Seven days later they were challenged with $10^5$ living M4

FIG. 2.—Number of lung nodules 14 days after injection of 2 × $10^4$ T3 fibrosarcoma cells into normal mice, or mice injected i.v. with 350 $\mu$g of CP or glucans 5 or 14 days previously.

FIG. 3.—Cytostatic macrophage activity (inhibition of RI leukaemia cell DNA synthesis) and non-specific anti-tumour activity (inhibition of lung nodules) expressed as percentage inhibition to show the temporal correlation between them.

cells in the contralateral footpad. Table III shows the proportion of mice developing tumours, and the size of the tumours expressed as average footpad diameter 30 days after challenge. No protection was conferred by irradiated M4 alone. The addition of 1 or 10 $\mu$g (but not 100 $\mu$g) CP resulted in complete protection. The relative inefficacy of larger doses of CP in combination with irradiated cells has been reported before (Scott, 1975; Bomford, 1975). Of the polysaccharides studied, only 100 $\mu$g lentinan

TABLE III.—*Growth of* $10^5$ *M4 Cells in Mice Injected 7 Days Previously with* $5 \times 10^5$ *Irradiated M4 Cell Alone, or Mixed with* C. parvum *or Polysaccharides*

| Treatment | | Footpad thickness 30 days after tumour challenge (mm ± s.e., n = 6) | Mice with tumours/total |
|---|---|---|---|
| Untreated controls | | 7·6 ± 0·8 | 6/6 |
| Irradiated M4 cells only | | 6·7 ± 0·6 | 6/6 |
| +CP | 1 μg | 2·1 ± 0·2* | 0/6 |
| | 10 μg | 2·0 ± 0·2* | 0/6 |
| | 100 μg | 4·6 ± 0·6 | 5/6 |
| +lentinan | 1 μg | 6·2 ± 0·6 | 6/6 |
| | 10 μg | 6·4 ± 0·5 | 6/6 |
| | 100 μg | 3·9 ± 0·9* | 3/6 |
| +pseudonigeran | 1 μg | 6·2 ± 0·6 | 6/6 |
| | 10 μg | 6·3 ± 0·6 | 6/6 |
| | 100 μg | 5·5 ± 0·5 | 6/6 |
| +dextran | 1 μg | 7·1 ± 0·5 | 6/6 |
| | 10 μg | 4·9 ± 0·6 | 5/6 |
| | 100 μg | 5·5 ± 0·4 | 6/6 |
| +DEAE-dextran | 1 μg | 5·3 ± 0·7 | 5/6 |
| | 10 μg | 7·9 ± 0·2 | 6/6 |
| | 100 μg | 6·6 ± 0·4 | 6/6 |
| +dextran sulphate | 1 μg | 7·4 ± 1·2 | 5/6 |
| | 10 μg | 7·0 ± 0·8 | 6/6 |
| | 100 μg | 3·8 ± 0·6* | 4/6 |

* Indicates $P < 0{\cdot}05$ relative to irradiated M4 only.

and dextran sulphate caused any significant inhibition of tumour growth. Even here, lentinan was not effective in a second experiment using 100, 200 or 400 μg mixed with $5 \times 10^5$ irradiated cells. The results with levans were uniformly negative and are omitted from Table III.

### DISCUSSION

The activities of the polysaccharides studied are summarized in Table IV. All induced cytostatic macrophages. Lentinan, yeast cell walls and pseudonigeran were also active in the lung-nodule inhibition test, which is considered to be mediated by a non-specific mechanism (Bomford and Olivotto, 1974). Although there is a temporal correlation between the presence of cytostatic macrophages in the peritoneal cavity and lung nodule inhibition after CP injection (Bomford and Olivotto, 1973), evidence is still awaited of a common effector basis for these phenomena. The parallel between the presence of cytostatic macrophages and lung nodule inhibition also held for lentinan, yeast walls and pseudonigeran in the present study, which further strengthens the case for suggesting a causal relationship between them.

Only lentinan and dextran sulphate amongst the polysaccharides displayed even marginal adjuvant activity for TSTA.

TABLE IV.—*A Summary of the Anti-tumour Activity of the Glucans and Fructosans*

| | Test | | | |
|---|---|---|---|---|
| | Macrophage activation | | | |
| Material | i.v. injection | i.p. injection | Lung nodule inhibition | Adjuvanticity TSTA |
| CP | +++ | +++ | +++ | +++ |
| Glucans | | | | |
| Yeast cell walls | ++ | ND | + | − |
| Lentinan | ++ | ND | + | ± |
| Pseudonigeran | ++ | ND | + | − |
| Dextran | − | + | − | − |
| DEAE-dextran | − | + | − | − |
| Dextran sulphate | − | + | − | ± |
| Fructosans | | | | |
| Levan | − | + | − | − |
| Carboxy-methyl-levan | − | + | − | − |

ND: Not done.

On the basis of the tests used in this study, therefore, it is concluded that the anti-tumour action induced by the polysaccharides is predominantly non-specific. We consider to what extent this conclusion is compatible with existing knowledge of the anti-tumour effects of these materials in other systems, and of their adjuvant properties.

Previous anti-tumour studies on lentinan (Chihara *et al.*, 1969, 1970), zymosan (Manowski *et al.*, 1957; Bradner *et al.*, 1958), dextrans (Ebbesen, 1974) and levan (Leibovici *et al.*, 1975) were all performed using the i.p. route of injection which, from the results of this study, might be expected to have induced cytostatic macrophages within the peritoneal cavity. The studies on zymosan and levan showed retardation of growth of transplantable tumours inoculated s.c. or i.p., but did not analyse its mechanism any further.

However, the anti-tumour effect of repeated i.p. administration of lentinan against the s.c. growth of Sarcoma 180 in mice (Chihara *et al.*, 1969, 1970) was not found in neonatally thymectomized mice (Maeda and Chihara, 1973). Although this might suggest that in this system lentinan stimulates specific immunity to TSTA, the data on the adjuvanticity of lentinan do not support this contention. Multiple i.p. injections of lentinan given after i.v. injection of sheep red blood cells (SRBC) stimulated the humoral response in normal, but not in T-cell-deprived mice (Dresser and Phillips, 1974), but it seems unlikely that i.p. lentinan would modify the humoral response to an s.c. tumour growth. Lentinan also failed to stimulate T-cell cytotoxicity in an allogeneic system of the DBA/2 P815 mastocytomas in C57BL/6 mice (Dennert and Tucker, 1973). An alternative explanation for the T-cell dependence of the anti-tumour effect of lentinan is that the simultaneous influence of cytostatic macrophages and a normal immune response is required, either alone being inadequate.

The effects of multiple i.p. injections of dextran, DEAE-dextran and dextran sulphate have been tested in two systems, the spontaneous leukaemia of AKR mice, and Rauscher leukaemia-virus-induced leukaemias in BALB/c mice (Ebbesen, 1974). If macrophages were effective in these systems, one might have predicted from the present results that all three dextrans should have shown some activity. However, in the AKR system dextran had no effect, DEAE-dextran improved survival, and dextran sulphate accelerated tumour development. In the BALB/c system, dextran and DEAE–dextran prolonged life when treatment started at the time of palpable spleen enlargement, whereas only dextran and dextran sulphate protected when injections started from the time of infection. Clearly factors other than cytostatic macrophages must be involved in these systems. It was suggested that the differential effects of the dextrans on the spread of virus or on the humoral response to it may play a role (Ebbesen, 1974).

Dextran sulphate (but not dextran) injected i.v. before i.v. immunization with SRBC potentiates the humoral response (Diamantstein *et al.*, 1971; Bradfield *et al.*, 1974). The failure of dextran sulphate to inhibitlung nodule formation in our studies is not surprising, as the mechanism does not involve an immune response to tumour cells (see above). Dextran sulphate potentiates killer T cells in the allogeneic P815 mastocytoma system (Vachek and Kolsch, 1975), which might explain why it showed a modest adjuvant effect for TSTA in the present study.

CP (McBride *et al.*, 1975), lentinan (Okuda *et al.*, 1972), dextran sulphate and levan (Pryjma, Humphrey and Klaus, 1974) all share the property of activating complement by the alternate pathway. Subsequently a plausible common mechanism for the induction of cytostatic macrophage by CP and polysaccharides has been provided by the finding of Schorlemmer, Davies and Allison (1976) that activated complement components induce lysosomal enzyme release from macrophages.

We thank Mrs S. Wishart and Mr N. Brown for excellent technical assistance, and Drs J. G. Howard and M. T. Scott for critical reading of the manuscript.

### REFERENCES

ADLAM, C. & SCOTT, M. T. (1973) Lympho-reticular Stimulatory Properties of *Corynebacterium parvum* and Related Bacteria. *J. med. Microbiol.*, **6**, 261.

BENACERRAF, B. & SEBESTYEN, M. M. (1957) Effects of Bacterial Endotoxins on the Reticuloendothelial System. *Fed. Proc.*, **16**, 860.

BIOZZI, G., HALPERN, B. N., BENACERRAF, B., STIFFEL, C. & MOUTON, D. (1956) Action de Certaines Polymeres Macromoléculaires et Notamment du Dextran et de la Polyvinylpyrrolidone sur la Fonction Phagocytaire du Système Reticuloendothelial. *C. r. Soc. Biol.*, **150**, 317.

BOMFORD, R. (1975) Active Specific Immunotherapy of Mouse Methylcholanthrene-induced Tumours with *Corynebacterium parvum* and Irradiated Tumour Cells. *Br. J. Cancer*, **32**, 551.

BOMFORD, R. & CHRISTIE, G. H. (1975) Mechanism of Macrophage Activation by *Corynebacterium parvum*. II. *In vivo* Experiments. *Cell. Immunol.*, **17**, 150.

BOMFORD, R. & OLIVOTTO, M. (1974) The Mechanism of Inhibition by *Corynebacterium parvum* of the Growth of Lung Nodules from Intravenously-injected Tumour Cells. *Int. J. Cancer*, **14**, 226.

BRADFIELD, J. W. B., SOUHAMI, R. L. & ADDISON, I. E. (1974) The Mechanism of the Adjuvant Effect of Dextran Sulphate. *Immunology*, **26**, 383.

BRADNER, W. T., CLARKE, D. A. & STOCK, C. C. (1958) Stimulation of Host Defence against Experimental Cancer. 1. Zymosan and Sarcoma 180 in Mice. *Cancer Res.*, **18**, 347.

CHIHARA, G., HAMURO, J., MAEDA, Y. Y., AVAI, Y. & FUKUOKA, F. (1970) Fractionation and Purification of the Polysaccharides with Marked Antitumour Activity, Especially Lentinan, from *Lentinus edodes* (Berk.) Sing. (an edible mushroom). *Cancer Res.*, **30**, 2776.

CHIHARA, G., MAEDA, Y. Y., HAMURO, J., SASAKI, T. & FUKOUKA, F. (1969) Inhibition of Mouse Sarcoma 180 by Polysaccharides from *Lentinus edodes* (Berk.) Sing. *Nature* (*Lond.*), **222**, 687.

DENNERT, G. & TUCKER, D. (1973) Antitumor Polysaccharide Lentinan—A T Cell Adjuvant. *J. natn. Cancer Inst.*, **51**, 1727.

DIAMANTSTEIN, T., WAGNER, B., BEYSE, I., ODENWALD, M. V. & SCHULZ, G. (1971) Stimulation of Humoral Antibody Formation by Polyanions. II. The Influence of Sulphate Esters of Polymers on the Immune Response in Mice. *Eur. J. Immunol.*, **1**, 340.

DRESSER, D. W. & PHILLIPS, J. M. (1974) The Orientation of the Adjuvant Activities of *Salmonella typhosa* Lipopolysaccharide and Lentinan. *Immunology*, **27**, 895.

EBBESEN, P. (1974) Influence of DEAE–dextran, Polybrene, Dextran and Dextran Sulphate on Spontaneous Leukaemia Development in AKR Mice and Virus Induced Leukaemia in BALB/c Mice. *Br. J. Cancer*, **30**, 68.

HALPERN, B. N., PREVOT, A.-R., BIOZZI, G., STIFFEL, C., MOUTON, D., MORARD, J. C., BOUTHILLIER, Y. & DECREUSEFOND, C. (1964) Stimulation de l'Activité Phagocytaire du Système Reticuloendothelial Provoquée par *Corynebacterium parvum*. *J. Reticuloendothelial Soc.*, **1**, 77.

HOUSTON, W. E., CRABBS, C. L., KREMER, R. J. & SPRINGER, J. W. (1976) Adjuvant Effects of Diethylaminoethyl–dextran. *Infect. Immun.*, **13**, 1559.

HOWARD, J. G., SCOTT, M. T. & CHRISTIE, G. H. (1973) Cellular Mechanisms underlying the Adjuvant Activity of *Corynebacterium parvum*: Interactions of Activated Macrophages with T and B Lymphocytes. In *Immunopotentiation*. Eds J. W. Wolstenholme and J. Knight. Ciba Foundation Symp., No. 18, 101, Amsterdam: Ass. Sci. Pub.

INMAN, J. K. (1975) Thymus-independent Antigens: the Preparation of Covalent, Hapten–Ficoll Conjugates. *J. Immunol.*, **114**, 704.

JOHNSTON, I. R. (1965) The Composition of the Cell Wall of *Aspergillus niger*. *Biochem. J.*, **96**, 651.

LEIBOVICI, J., SINAI, Y., WOLMAN, M. & DAVIDAI, G. (1975) Effects of High-molecular Levan on the Growth and Spread of Lymphoma in AKR Mice. *Cancer Res.*, **35**, 1921.

MAEDA, Y. Y. & CHIHARA, G. (1973) The Effects of Neonatal Thymectomy on the Antitumour Activity of Lentinan, Carboxymethylpachymaran and Zymosan, and their Effects on Various Immune Responses. *Int. J. Cancer*, **11**, 153.

MANOWSKI, Z. T., YAMASHITA, M. & DILLER, I. C. (1957) Effect of *Candida guilliermondi* Polysaccharide on Transplantable Mouse Sarcoma 37. *Proc. Soc. exp. Biol. Med.*, **96**, 79.

MCBRIDE, W. H., WEIR, D. M., KAY, A. B., PEARCE, D. & CALDWELL, J. R. (1975) Activation of the Classical and Alternate Pathways of Complement by *Corynebacterium parvum*. *Clin. exp. Immunol.*, **19**, 143.

MORENO, C., COURTENAY, B. M. & HOWARD, J. G. (1976) Molecular Size and Structure in Relation To the Tolerogenicity of Small Fructosans (Levans). *Immunochemistry*, **13**, 429.

OKUDA, T., YOSHIOKA, T., IKEKAWA, G., CHIHARA, G. & NISHIOKA, K. (1972) Anti-complementary Activity of Anti-tumor Polysaccharides. *Nature, New Biol.*, **238**, 59.

OLIVOTTO, M. & BOMFORD, R. (1974) *In vitro* Inhibition of Tumour Cell Growth and DNA Synthesis by Peritoneal and Lung Macrophages from Mice Injected with *Corynebacterium parvum*. *Int. J. Cancer*, **13**, 478.

PRYJMA, J., HUMPHREY, J. H. & KLAUS, G. G. B. (1974) C3 Activation and T-independent B Cell Stimulation. *Nature, Lond.*, **252**, 505.

RIGGI, S. J. & DI LUZIO, N. R. (1961) Identification of a Reticuloendothelial Stimulating Agent in Zymosan. *Am. J. Physiol.*, **200**, 297.

SCHORLEMMER, H., DAVIES, P. & ALLISON, A. C. (1976) Ability of Activated Complement Components to Induce Lysosomal Enzyme Release from Macrophages. *Nature, Lond.*, **261**, 48.

SCOTT, M. T. (1974*a*) *Corynebacterium parvum* as an Immunotherapeutic Anticancer Agent. *Seminars Oncology*, **1**, 367.

SCOTT, M. T. (1974*b*) *Corynebacterium parvum* as a Therapeutic Antitumor Agent in Mice. 1. Systemic Effects from Intravenous Injection. *J. natn. Cancer Inst.*, **53**, 855.

SCOTT, M. T. (1975) Potentiation of the Tumor-specific Immune Response by *Corynebacterium parvum*. *J. natn. Cancer Inst.*, **55**, 65.

VACHEK, H. & KÖLSCH, E. (1975) Dextran Sulphate Stimulates the Induction but Inhibits the Effector Phase in T Cell-mediated Cytotoxicity. *Trans-Plantation*, **19**, 183.

WITTMAN, G. (1970) The Use of Diethylaminoethyl-Dextran (DEAE-D) as Adjuvant for Immunization of Guinea-pigs with Inactivated Foot-and-Mouth Disease (FMD) Virus. *Z. Bakt. Parasit. Infect. skr. Hyg.*, 1. Orig. **213**, 1.

WOODRUFF, M., DUNBAR, N. & GHAFFAR, A. (1973) The Growth of Tumours in T-cell-deprived Mice and their Response to Treatment with *Corynebacterium parvum*. *Proc. R. Soc. Lond. B.*, **184**, 97.